# Cannabinoid receptor 1 expression is higher in muscle of old vs. young males, and increases upon resistance exercise in older adults

**DOI:** 10.1038/s41598-021-97859-3

**Published:** 2021-09-15

**Authors:** Sebastiaan Dalle, Katrien Koppo

**Affiliations:** Exercise Physiology Research Group, Department of Movement Sciences, KU Leuven, Tervuursevest 101, 3001 Leuven, Belgium

**Keywords:** Ageing, Physiology, Cell biology, Lipid signalling

## Abstract

Aged skeletal muscle undergoes metabolic and structural alterations eventually resulting in a loss of muscle strength and mass, i.e. age-related sarcopenia. Therefore, novel targets for muscle growth purposes in elderly are needed. Here, we explored the role of the cannabinoid system in muscle plasticity through the expression of muscle cannabinoid receptors (CBs) in young and old humans. The CB_1_ expression was higher (+ 25%; p = 0.04) in muscle of old (≥ 65 years) vs. young adults (20–27 years), whereas CB_2_ was not differently expressed. Furthermore, resistance exercise tended to increase the CB_1_ (+ 11%; p = 0.055) and CB_2_ (+ 37%; p = 0.066) expression in muscle of older adults. Interestingly, increases in the expression of CB_2_ following resistance exercise positively correlated with changes in key mechanisms of muscle homeostasis, such as catabolism (FOXO3a) and regenerative capacity (Pax7, MyoD). This study for the first time shows that CB_1_ is differentially expressed with aging and that changes in CB_2_ expression upon resistance exercise training correlate with changes in mediators that play a central role in muscle plasticity. These data confirm earlier work in cells and mice showing that the cannabinoid system might orchestrate muscle growth, which is an incentive to further explore CB-based strategies that might counteract sarcopenia.

## Introduction

Ageing is associated with changes that eventually affect health (span). Skeletal muscle is not only key for mobility, but also fulfills central metabolic functions, such as substrate storage and oxidation. With advancing age, there is a gradual decline in muscle strength and muscle mass, also referred to as sarcopenia^1^. This condition decreases the quality of life and health through an increased risk for immobility, social isolation and metabolic conditions such as type 2 diabetes.

Currently, resistance exercise (RE), whether or not combined with protein^2^, omega-3^3,4^ or vitamin D^5^ supplementation, is considered the primary intervention to attenuate age-related muscle deterioration. However, muscular adaptations to RE are less pronounced in old compared to young adults^6,7^. Hence, it remains important to better understand which molecular networks underlie muscular degeneration with advancing age to eventually develop novel (adjuvant) therapies that can attenuate a loss in muscle strength and muscle mass.

Multiple factors contribute to age-related muscle wasting, e.g. inflammation^8,9^, malnutrition^10^, metabolic dysregulations^11^ and physical inactivity^12^. Recently, there is a growing interest in the role of the cannabinoid system in controlling plasticity in metabolic tissues, such as adipose tissue and skeletal muscle^13–15^. For example, increased endocannabinoid activity (e.g. high tissue levels of the endocannabinoids) has been associated with metabolic dysregulation such as obesity^16,17^. The cannabinoid system is composed of endocannabinoids, e.g. anandamide and 2-arachidonoylglycerol (2-AG), which are lipid mediators binding to the cannabinoid receptor type 1 (CB_1_) and CB_2_. These CBs are expressed in many tissues, and most ubiquitously in the nervous and immune system, which might explain the central role of the cannabinoid system in neural processes and in the inflammatory signaling.

CBs, as G-coupled protein receptors, induce intracellular signaling through G proteins which in turn induce or inhibit second messengers and intermediates, including cyclic adenosine monophosphate, phosphatidylinositide-3-kinase (PI3K) and phospholipase A and C^18^. In neural cells, CB activation induces MAPK signaling which regulates many cellular processes including proliferation, differentiation, apoptosis and energy metabolism. Whereas the signaling responses upon CB binding are well described in neural tissues, it is not understood how CB_1/2_ affect (MAPK-mediated) downstream signaling in skeletal muscle tissue. Nevertheless, recent developments in mice indicate that CB signaling interferes with muscle metabolism^19^, muscle maintenance^15^ and regenerative processes^13^.

CB_1_ antagonism in mice reversed age-related metabolic dysregulations^19,20^ and improved muscle regeneration in a murine model of Duchenne muscular dystrophy^14^. Moreover, CB_1_ knock-out in skeletal muscle protected old mice from insulin resistance and upregulated intermediates of the mTORC1 pathway, responsible for muscle protein synthesis (MPS)^15^. In vitro data confirmed that CB_1_ inhibition stimulated mTORC1 and MPS in human myotubes^21^. Besides anabolic signaling, also muscle catabolism plays a crucial role in muscle maintenance. Unfortunately, there are no studies that investigated whether modifications in the cannabinoid tone affect catabolic mechanisms, such as the ubiquitin–proteasome system (e.g. FOXO and MuRF1) or autophagy (e.g. LC3b). Unpublished data from our lab showed that CB_2_ was downregulated during muscle atrophying conditions such as unloading and CTX-induced injury^22^ in mice (Supplementary Figure 1). Others demonstrated that CB_2_ agonism improved, whereas CB_2_ antagonism impaired, muscle regeneration^23–25^.

Despite these promising data indicating that CBs are interesting candidates to target (age-related) muscle wasting, little is known about the expression and modulation of CBs in human skeletal muscle tissue. In anticipation of CB (ant)agonists approval for human application, the present study explores for the first time muscle CB_1_ and CB_2_ expression in different age groups and in response to RE. We hypothesize that CB_1_ is more expressed in muscle of old vs. young males, indicative for increased endocannabinoid activity that might be associated with age-related metabolic dysregulations^19,20^. No females were included since hormonal fluctuations in young females might interfere with the cannabinoid signaling. Furthermore, we hypothesize that RE downregulates CB_1_ and upregulates CB_2_ expression in muscle tissue of old adults (males and females), indicating an exercise-induced improvement in metabolic health and an improved myogenicity, respectively. Finally, we investigate whether responses in CB expression are related to anabolic, catabolic and regenerative muscle markers, and whether they occur in a fiber-specific way.

## Results

### Experiment 1 (Fig. 1)

**Figure 1:**
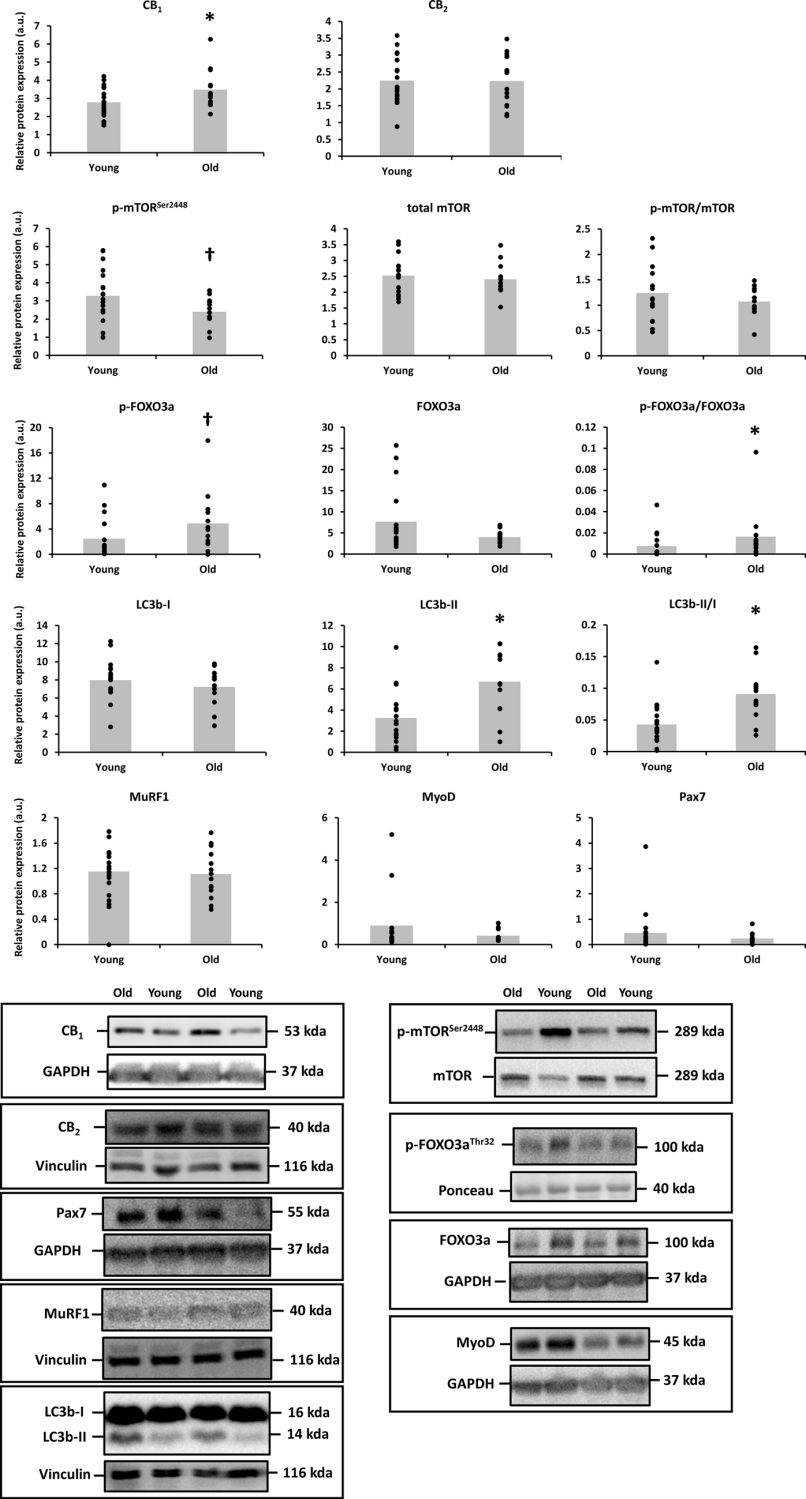
Differences in the protein expression of cannabinoid receptor 1 (CB_1_), CB_2_ and phospho and total mammalian target of rapamycin (mTOR) at Ser2448, phospho and total forkhead box O (FOXO)3a at Thr32, microtubule-associated protein 1A/1B-light chain 3 (LC3b), muscle RING finger-1 (MuRF1), MyoD and Pax7 between skeletal muscle of young (n = 18) and old adults (n = 14). Protein targets were normalized to glyceraldehyde 3-phosphate dehydrogenase (GAPDH), vinculin or the total protein form. Data are shown as mean (bar graph in grey) with individual data points (black dots superimposed on the bar graphs). *p < 0.05 and ^†^p = 0.05–0.10 for Independent-Samples *t* Test or Mann–Whitney *U* Test. Raw western blot data of all replicates are displayed in Supplementary Figure 2. Ponceau red staining of the presented western blots are included in Supplementary Figure 3.

Skeletal muscle CB_1_ expression was higher in healthy, old adults (65–84 years) compared to young adults (20–27 years; + 25%; Cohen’s d = 0.73; p = 0.045), whereas CB_2_ was not differently expressed between age groups.

Phospho-mTOR^Ser2448^, but not phospho/total mTOR^Ser2448^ (p = 0.337), tended to be less expressed in skeletal muscle of old vs. young adults (− 27%; d = 0.39; p = 0.066). Expression of the catabolic marker phospho/total FOXO3a^Thr32^ was higher in old vs. young skeletal muscle (+ 140%; d = 1.96; p = 0.032), whereas its downstream mediator, the atrogene MuRF1, was not different between both age groups (p = 0.752). The autophagy markers LC3b-II (d = 1.21; p = 0.002) and LC3b-II/I (d = 1.30; p = 0.001) were ~ 2-fold higher in older adults compared to younger adults. Finally, myogenic markers Pax7 and MyoD were not differentially expressed in the muscle of both age groups.

The correlation between the expression of both CB_1_ and CB_2_, and the expression of anabolic, catabolic and myogenic markers in the pooled dataset of young and old adults revealed no significant correlations.

### Experiment 2

The 12-week RE program in healthy, old adults (65–78 years) increased the 1 repetition maximal (1RM) leg press strength from 175.8 ± 16.3 to 222.1 ± 19.1 kg (+ 31 ± 6%; d = 0.60; p < 0.001) and muscle density from 49.9 ± 0.6 to 51.1 ± 0.5 HU (+ 2.5 ± 1.1%; d = 0.47; p = 0.033) but not upper leg muscle volume (from 516.9 ± 31.6 to 518.3 ± 30.5 cm^3^; p = 0.838) (Supplementary Figure 6).

**Figure 2:**
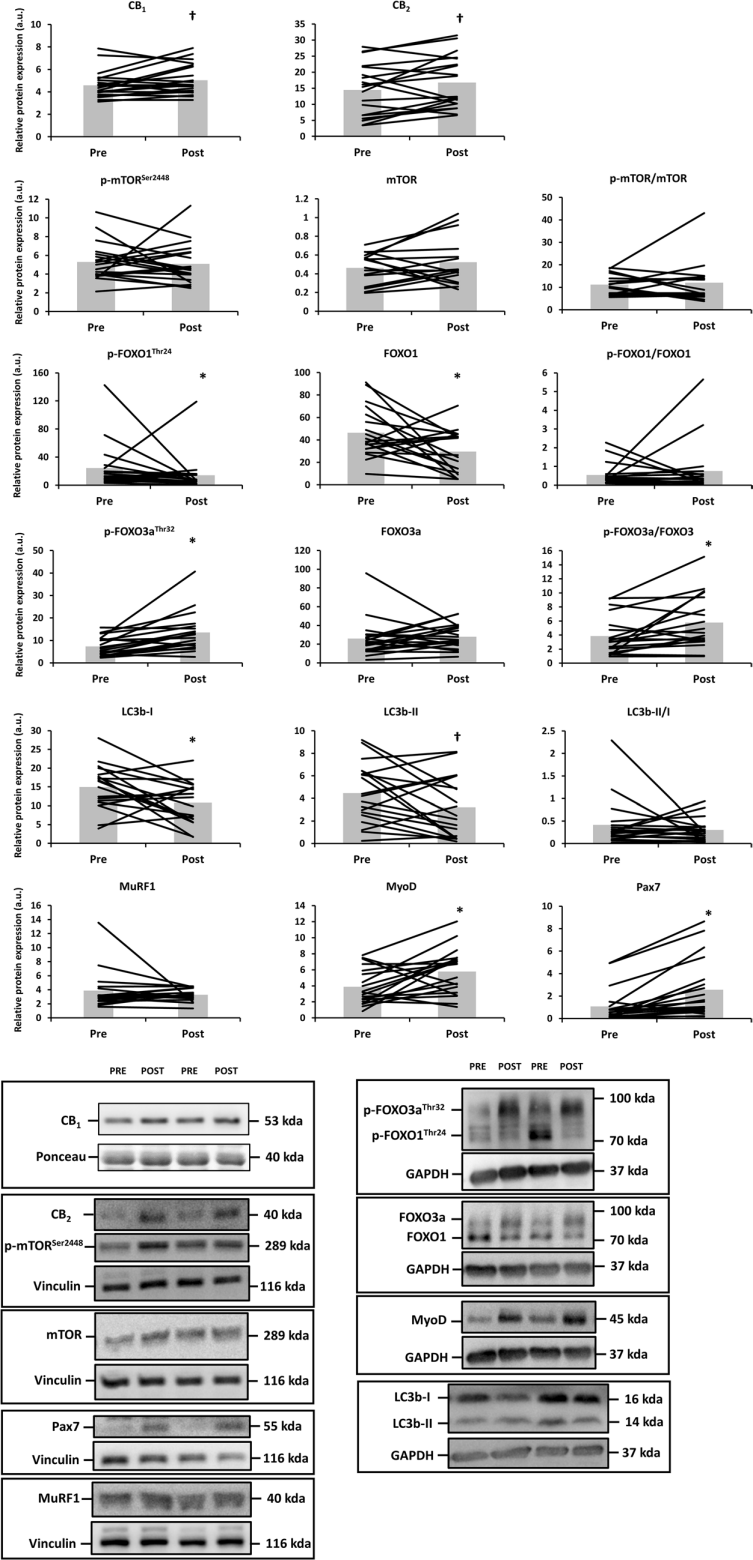
Differences in the protein expression of cannabinoid receptor 1 (CB_1_), CB_2_, phospho and total mammalian target of rapamycin (mTOR) at Ser2448, phospho and total forkhead box O (FOXO)1/3a at Thr24/32, microtubule-associated protein 1A/1B-light chain 3 (LC3b), muscle RING finger-1 (MuRF1), MyoD and Pax7 before (Pre) and after (Post) resistance exercise in old adults (n = 19). Protein targets were normalized to glyceraldehyde 3-phosphate dehydrogenase (GAPDH), vinculin or the total protein form. Data are shown as mean (bar graph in grey) with individual data points (black lines superimposed on the bar graphs). *p < 0.05 and ^**†**^p = 0.05–0.10 for Paired-Samples *t* Test or Wilcoxon signed-rank test. Raw western blot data of all replicates are displayed in Supplementary Figure 4. Ponceau red staining of the presented western blots are included in Supplementary Figure 5.

Skeletal muscle CB_1_ (+ 11 ± 5%; d = 0.36; p = 0.055) and CB_2_ expression (+ 37 ± 15%; d = 0.28; p = 0.066) tended to increase upon RE in old adults (Fig. 2). Similarly, the RE protocol increased the muscle proteolytic marker p-FOXO3a^Thr32^ (+ 105 ± 22%; d = 0.91; p = 0.002), but not total FOXO3a, and decreased p-FOXO1^Thr24^ (− 4 ± 29%; d = 0.34; p = 0.016) and total FOXO1 (− 23 ± 14%; d = 0.81; p = 0.025). RE also decreased the muscle expression of autophagy markers LC3b-I (d = 0.73; p = 0.023) and LC3b-II (d = 0.47; p = 0.065) with ~ 10%. Yet, the LC3b-II/I ratio was unaffected due to RE. Finally, the expression of myogenicity markers MyoD and Pax7 was upregulated after RE (+ 108 ± 46%; d = 0.73; p = 0.016 and + 4950 ± 4626%; d = 0.70; p < 0.001), whereas phospho/total mTOR^Ser2448^ remained unaffected by RE in old adults.

**Figure 3:**
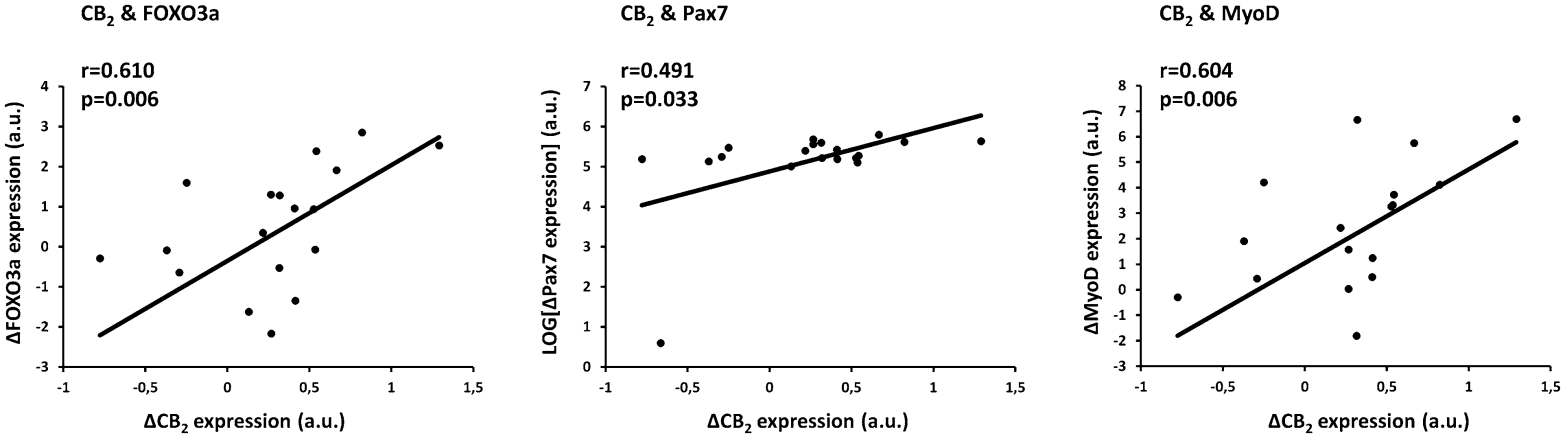
Pearson (normal data) or Spearman (non-normal data) correlations between the change (Δ; Post–Pre RE) in CB_2_ expression and the change in forkhead box O 3a (FOXO3a), MyoD and Pax7 expression. Non-normal data are presented following ^10^log-transformation.

The change in expression (Post minus Pre RE) of the CB_2_ was associated with the change in expression in these central markers of muscle maintenance (Fig. 3). Pearson correlations revealed that CB_2_ correlated with total FOXO3a (r = 0.610; p = 0.006) and with both myogenic markers MyoD (r = 0.604; p = 0.006) and Pax7 (r = 0.491; p = 0.033), whereas CB_1_ was not related to any of the markers of muscle maintenance.

**Figure 4:**
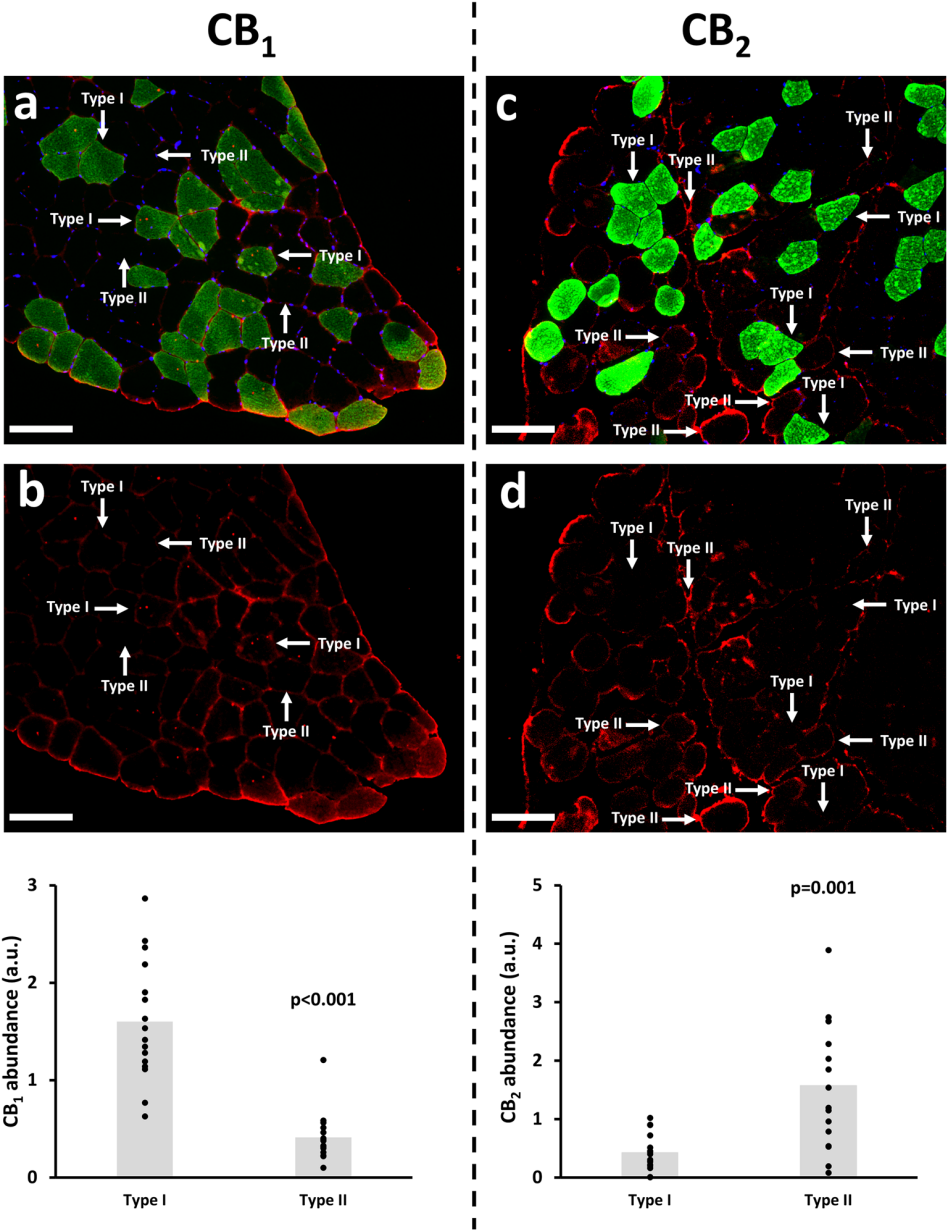
Representative muscle cryosections (*m. Vastus Lateralis*) stained for MyHC I (type I fibers; green), Hoechst (nuclei; blue), CB_1_ (Red, panel **a**,**b**) or CB_2_ (Red, panel **c**,**d**). CB_1_ and CB_2_ expression was quantified in a fiber type-specific way (type I vs. type II fibers), in 16 muscle cryosections. Scale bar: 50 µm.

As expected, CB_1_ and CB_2_ were highly expressed in the sarcolemma. The expression of CB_1_ in the *m. Vastus Lateralis* of older adults was more pronounced in type I vs. type II fibers (+ 288%; p < 0.001), whereas CB_2_ was more expressed in type II vs. type I fibers (+ 268%; p = 0.001) (Fig. 4).

## Discussion

Recently, it was shown in mice that CBs are key players in the regulation of myogenicity and muscle metabolism, both features that are affected with advancing age, and that contribute to sarcopenia. Consequently, it has been suggested that CB (ant)agonism is a promising strategy to counteract muscle degeneration, and more specifically age-related sarcopenia^15^. Unfortunately, CB modulation is not yet feasible in humans. Hence, the present explorative study for the first time investigates whether CB expression is different in old vs. young skeletal muscle tissue, and whether CB expression is responsive to anabolic stimuli such as RE.

The findings of the present study demonstrate that CB_1_ is differently expressed in skeletal muscle of young (20–27 years) vs. old adults (65–84 years), and that both CB_1_ and CB_2_ expression tend to increase upon chronic RE in old adults. Interestingly, the RE-induced change in CB_2_ expression was associated with changes in the catabolic marker FOXO3a and with changes in markers of myogenicity, i.e. MyoD and Pax7. More interventional work in animals and humans is needed to unravel the mechanisms via which CBs might affect muscle plasticity in response to age and exercise.

It was reported that the cannabinoid tone (e.g. circulating AEA and 2-AG levels) was typically higher in metabolic conditions, such as obesity^17,26^, and that increased CB_1_ expression related to a decreased metabolic health, as some^27,28^, but not all^17,29^, researchers observed higher CB_1_ expression levels in the adipose tissue of overweight/obese adults when compared to lean. Furthermore, the cannabinoid tone positively correlated with (markers of) adiposity and insulin resistance^26,29^ and CB_1_ expression in visceral adipose tissue positively correlated with BMI^27^. Therefore, in the present study, it is conceivable that the increased CB_1_ expression in the muscle of older vs. young adults reflects an increased cannabinoid tone and decreased metabolic health in the older adults. Although the older participants did not suffer from explicit metabolic conditions, it is very likely that their metabolic health is generally lower when compared to physically active, young adults (which might be reflected by their higher BMI, i.e. 27.2 in the older participants vs. 23.8 kg·m^−2^ in the young participants). Also, half of the older adults used statins to lower increased cholesterol levels.

The effect of age on CB_1_ expression has previously been studied in mice, be it on the gene and not on the protein expression level. Evidence in mice was equivocal. CB_1_ expression was lower in the m. *Gastrocnemius* of 1 year vs. 2 weeks old mice^30^, whereas others reported a higher CB_1_ expression in the m. *Gastrocnemius* of 16-month old vs. 4-months old mice^19^. This latter observation is in accordance with our observation that CB_1_ expression was higher in old vs. young adults. Besides metabolic health, it remains unclear which behavioral (e.g. physical activity) and/or biological (e.g. hormone levels) changes contribute to the effect of ageing on muscle CB_1_ expression.

Whereas acute exercise upregulates circulating endocannabinoid levels (particularly AEA), the effect of chronic exercise on the endocannabinoid tone and on muscle CB expression remains poorly understood. We hypothesized that muscle CB_1_ expression would decrease in response to a 12-weeks RE program, reflecting an exercise-induced improvement in metabolic health. Surprisingly, CB_1_ expression increased following RE. This observation is very unlikely to reflect a deterioration in metabolic health, which is why alternative mechanisms might be at work. One hypothesis is that the increased CB expression can be explained by a change in the muscle fiber type composition since CB_1_ and CB_2_ expression was more pronounced in type I and type II muscle fibers, respectively. However, muscle MyHC type I and II expression did not alter due to RE, and if any changes were to be expected following RE, it would rather be an increase in type II fibers, which would have resulted in a relative decrease in CB_1_ expression rather than an increase. Alternatively, it is possible that an RE-induced increase in mitochondrial density^31^ explains the increases in CB_1_, as the receptor is highly expressed in mitochondria of human muscle tissue^32^. However, in contrast to endurance training, it remains debatable whether high-load RE increases the mitochondrial content^33^. Finally, RE-induced changes in the relative contribution and/or signaling of muscle-resident non-myogenic cell types^34,35^ such as immune cells, vascular cells and neural cells might also explain part of the increase in CB_1_ expression.

Similar to CB_1_, also CB_2_ expression tended to be increased upon RT. Pharmaceutical CB_2_ agonism increased the expression of satellite cell growth markers such as MyoD and myogenin^23^, while CB_2_ knock-out decreased MyoD and myogenin expression during murine muscle regeneration^13^. Therefore, the increased CB_2_ expression in the present study might reflect an improved myogenic capacity, e.g. satellite cell content, in response to RE. Indeed, MyoD and Pax7 expression significantly increased upon RE, and the increase of both markers positively correlated with the increase in CB_2_ expression. Together, these data indicate that there might be regulatory networks that relate CB_2_ biology to satellite cell myogenicity. However, there is need for more evidence to explore a potential association between muscle progenitor cells and CBs, especially in humans.

Whereas evidence in mice^30^ and frogs^36^ showed that CB_1_ gene expression was higher in predominantly fast (e.g. *Gastrocnemius*, *Tibialis Anterior*) vs. slow (e.g. *Soleus*) muscles, our data show that CB_1_ expression was more highly abundant in type I than in type II fibers of the m. *Vastus Lateralis* of older adults (based on colorimetric analysis of individual muscle fibers). From a metabolic perspective, it seems indeed more logical that CB_1_, which is an important regulatory player in peripheral oxidative capacity^32^, is more abundant in type I fibers, which exhibit a higher oxidative capacity than type II fibers.

In conclusion, cell culture and murine experiments suggested that CBs can be a promising target to treat cachexia and sarcopenia through modulation of the metabolism and muscle regenerative capacity. Yet, the role of CB modulation as a window of opportunity to treat muscle devastating conditions in (old) humans remains unstudied. The present explorative study for the first time shows that CB_1_ is differentially expressed in muscle of old (i.e. higher expression) vs. young (i.e. lower expression) adults, and that both CB_1_ and CB_2_ expression increases upon RE, the most important anabolic intervention for muscle growth purposes. Interestingly, RE-induced increases in CB_2_ correlated with markers of myogenicity (MyoD and Pax7). These data imply that CB modulation might be a promising tool to combat muscle degeneration. As a next step, human intervention studies should be performed to confirm whether CBs are promising targets to improve the muscle phenotype in age-related sarcopenia and/or muscle devastating conditions, such as cancer cachexia.

## Materials and methods

### Experiment 1 (young vs. old adults)

Study participants were 18 Caucasian young males (according to their biological sex) (age: 24 (20–27) years; height: 1.83 (1.72–1.91) m; body mass: 77 (71–86) kg; BMI: 23.8 (22.1–26.4) kg·m^−2^) and 14 community-dwelling, non-sarcopenic (cfr. EWGSOP2^1^) Caucasian old males (according to their biological sex) (age: 70.8 (65–78) years; height: 1.74 (1.70–1.80) m; body mass: 82 (71–99) kg; BMI: 27.2 (24.3–31.1) kg·m^−2^). All participants were recreationally active, but did not engage in a consistent training program or any sport at a competitive level. Muscle tissues were collected in previously executed studies at our laboratory (2014–2019; Exercise Physiology Research Group; KU Leuven; Belgium) and have been stored at − 80 °C immediately after completion of the study. The only inclusion criteria for selection were age (< 30 years or ≥ 65 years, respectively) and sufficiently muscle tissue available (≥ 15 mg). Exclusion criteria were BMI < 20 or > 35 kg·m^−2^, unstable body weight (i.e. 2-kg change during the past 6 months), smoking, disease (cancer, liver, renal, musculoskeletal, neurodegenerative or unstable cardiovascular dysfunctions), smoking or supplementation of cannabis or cannabis-related products (e.g. CBD oil), regular protein supplementation and structural RE or endurance training. None of the young participants took any medication. The medicine intake of the older participants reflected the real-life situation, e.g. a large proportion of the older participants took blood thinners (n = 4; 29%; e.g. asaflow), statins (n = 7; 50%; e.g. simvastatin), beta blockers (n = 7; 50%; e.g. bisoprolol), antacids (n = 3; 21%; e.g. nexiam) and medication to treat osteoporosis (n = 1; 7%) and to treat benign prostatic hyperplasia (n = 2; 14%). All muscle biopsies were sampled by the same MD, and all samples have been prepared simultaneously (August 2020) for analyses by the same researcher. This study was approved by the Ethics Committee Research UZ/KU Leuven (S58361, S61809) and conformed to the Declaration of Helsinki. All participants gave their written informed consents.

### Experiment 2 (resistance exercise in old adults)

Study participants were 19 physically active, non-sarcopenic, Caucasian old adults (of which 7 females according to their biological sex) (age: 70.3 (65–78) years; height: 1.68 (1.49–1.80) m; body mass: 76 (62–99) kg; BMI: 26.8 (23.3–31.1) kg·m^−2^) with the same exclusion criteria as reported in experiment 1. The medicine intake of the older participants reflected the real-life situation, e.g. a large proportion of the older participants took blood thinners (n = 6; 32%; e.g. assaflow), statins (n = 9; 47%; e.g. simvastatin), beta blockers (n = 7; 37%; e.g. bisoprolol), antacids (n = 4; 21%; e.g. nexiam), antidepressants (n = 1; 5%), sleep medication (n = 1; 5%), and medication against migraine (n = 2; 11%), to treat osteoporosis (n = 2; 11%) and to treat benign prostatic hyperplasia (n = 1; 5%). Muscle biopsies were sampled before (Pre) and after (Post) 12-weeks RE program. All biopsies were taken at least 72 h after RE to exclude interference of acute exercise on muscle molecular signaling^37^. Three supervised training sessions per week started with a 10-min warm-up on a cycle ergometer, followed by lower extremity exercises (leg press, leg extension and calf raises). In the first 6 weeks, 2 sets of 12–15 repetitions at 70% of the 1-repetition maximum (1RM) were performed, and in the last 6 weeks, participants completed 3 sets of 10–12 repetitions at 80% of the 1RM. This study was approved by the Ethics Committee Research UZ/KU Leuven (S61809) and conformed to the Declaration of Helsinki. All participants gave their written informed consents.

### Muscle biopsy procedure

Prior to muscle biopsies, fasted participants reported at the lab and rested for 45 min. A needle biopsy (~ 150 mg) of the *m. vastus lateralis* was performed under local anaesthesia (2% xylocaine, 1 mL subcutaneously) with a 5-mm Bergström-type needle at the Pre and Post experimental session. The muscle sample was immediately frozen in liquid nitrogen and stored at − 80 °C for later biochemical analyses.

### Protein extraction and western blot analysis

As previously described^3,22,38^, frozen skeletal muscle tissue (15–20 mg) was homogenized (4 × 20 s) in ice-cold lysis buffer (1/10 w/v; 50 mM Tris–HCl, pH 7.0; 270 mM sucrose; 5 mM EGTA; 1 mM EDTA; 1 mM sodium orthovanadate; 50 mM glycerophosphate; 5 mM sodium pyrophosphate; 50 mM sodium fluoride; 1 mM dithiothreitol; 0.1% Triton X-100; and a complete protease inhibitor tablet) using the FastPrep (MP Biomedicals, Santa Ana, CA). The obtained homogenates were centrifuged at 10,000*g* (20 min at 4 °C) and the supernatant was stored at − 80 °C. Protein concentrations were assessed with the DC protein assay kit using a BSA protein standard (Bio-Rad Laboratories, Nazareth, Belgium), and protein concentrations were equalized by adding lysis buffer. Eventually, laemmli was added (20% of the total volume) to obtain muscle lysates. Proteins (20–35 µg) were separated by SDS-PAGE (8–12% gels) and transferred to polyvinylidene difluoride membranes. Then, membranes were blocked in TBS-T (tris-buffered saline with Tween-20) + 5% BSA for 1 h. Overnight, membranes were incubated at 4 °C in TBS-T + 5% BSA with the following antibodies: p-mammalian target of rapamycin (p-mTOR, CST-2971S, 1/1000, 289 kDa), total mTOR (CST-2983S, 1/1000, 289 kDa), CB_1_ (ab259323, 1/400, 53 kDa), CB_2_ (ab3561, 1/400, 40 kDa), Vinculin (V9131, 1/2000, 116 kDa), Pax-7 (DSHB, 1/300, 55 kDa), MyoD (sc-377460, 1/400, 45 kDa), muscle RING finger-1 (MuRF1, sc-2920, 1/500, 40 kDa), microtubule-associated protein 1A/1B-light chain 3 (LC3b, CST- 2775S, 1/1000, 14–16 kDa), glyceraldehyde 3-phosphate dehydrogenase (GAPDH, CST-2118S, 1/5000, 37 kDa), p-forkhead box O (FOXO) 1^Thr24^/3a^Thr32^ (CST-9464S, 1/500, 705–100 kDa) and FOXO3a (CST-2497S, 1/500, 70–100 kDa). Hereafter, membranes were incubated at room temperature for 45 min with horseradish peroxidase-conjugated secondary antibodies (1/7000; Sigma Aldrich, Bornem, Belgium). Membranes were scanned and quantified with Genesnap and Genetools Softwares (Syngene, Cambridge, UK). Protein targets were normalized to glyceraldehyde 3-phosphate dehydrogenase (GAPDH), vinculin or the total form. Staining with ponceau red staining confirm homogenous sample loading. Ponceau red staining of the representative lanes of the western blot analyses presented in Figs. 1 and 2 are displayed in Supplementary Figure 3 and Supplementary Figure 5, respectively. The full-length western blot membrane outputs of Figs. 1 and 2 are displayed in Supplementary Figure 2 and Supplementary Figure 4, respectively.

### Immunohistochemistry

Muscle tissues were embedded in tissue freezing medium (Leica Biosystems, Wetzlar, Germany) and frozen in liquid nitrogen-cooled isopentane. Serial cryosections (7 µm thick) were cut with a cryostat (Leica Biosystems CM1850, Wetzlar, Germany) at − 20 °C. Prior to histological analyses, cryosections were thawed at room temperature (RT), washed with PBS and fixed with acetone at − 20 °C. Next, cryosections were blocked for 1 h in PBS containing 2% bovine serum albumin (BSA) and incubated overnight at 4 °C in a humid chamber with the following primary antibodies: BA-F8 (1/200, myosin heavy chain (MyHC) I, DSHB, Iowa), CB_1_ (1/200, ab23703) or CB_2_ (1/200) dissolved in PBS. Hereafter, the conjugated secondary antibodies were applied for 1 h at 37 °C in a humid chamber: goat anti-mouse Alexa-488 IgG2 (1/300, MyHC I) and goat anti-rabbit Alexa-594 IgG (1/300, CB_1_ and CB_2_). Next, cryosections were washed with PBS and for 15 min incubated with Hoechst (1 µg·mL^−1^) at room temperature. Sections were mounted with Dako fluorescence mounting medium (Dako, S3023) and visualized by fluorescence microscopy (Nikon E1000, Germany). The epifluorescence signal was recorded with FITC (MyHC I), DAPI (Hoechst) and Texas Red (CB_1_ and CB_2_) excitation filters. CB_1_ and CB_2_ expression was quantified in 16 muscle cryosections. Six distinct regions within each cryosection were analysed for CB_1/2_ expression, i.e. 3 regions with type I muscle fibers (MyHC I^+^ fibers) and 3 regions with type II muscle fibers (MyHC I^−^ fibers). For both receptors, regions with type I and type II muscle fibers were selected in the muscle cryosections with ImageJ 1.52a (National Institutes of Health, Bethesda, USA). Next, the blue (cell nuclei) and green (MyHC I) signal was removed, and the red signal (CB_1_ and CB_2_ expression) was quantified in a fiber-type specific way.

### Muscle strength

The 1-RM was measured on the leg press device as previously described^39^. Shortly, participants started with a warm-up set of 8 repetitions at ~ 50% of the estimated 1-RM, followed by a set of 3 repetitions at ~ 70% of the estimated 1-RM. Subsequent lifts were single repetitions with progressively heavier resistances until failure. A 2-min recovery period was provided between each attempt. The heaviest successful lift was determined as 1-RM. Participants were familiarized with the 1-RM leg press procedure. The 1-RM was assessed at baseline (PRE), after 6 weeks of RE and after 12 weeks of RE (POST).

### Muscle volume and density

As previously reported^3^, a computed tomography scan (Somatom Force^®^, Siemens Medical Solutions, Erlangen, Germany) was used to measure the muscle volume of the left upper leg. Four thick axial images of 5 mm were obtained at the midpoint of the distance between the medial edge of the trochanter major and the intercondyloid fossa of the femur. Standard Hounsfield units (HU) ranges for muscle (0–100) were used to segment muscle area, and were corrected for bone marrow. The 4 slices were put together as one slice of 20 mm. Total muscle volume was determined by an expert radiologist, with a software program developed at the University Hospital.

### Statistics

Data are presented as mean ± SEM or mean (range), and were tested for normality with a Kolmogorov–Smirnov test. Conditions were compared with a two-tailed Independent-Sample *t* Test or Mann–Whitney *U* Test (experiment 1), or a two-tailed Pair-Sampled *t* Test or Wilcoxon signed-rank test (experiment 2) (SPSS 20, IBM, Chicago, IL). Where relevant, Cohen’s D (d) was calculated as an index of effect size (0.2 = small, 0.5 = medium, > 0.8 = large). Correlations were assessed by the Pearson (normal data) or Spearman (non-normal data) correlation coefficient analyses (experiment 2, SPSS 20). Significance (*) was accepted at p < 0.05 and trends (^†^) were set at p = 0.05–0.10.

## Supplementary Information

Supplementary Figures.

## Data Availability

The datasets generated during and/or analysed during the current study are available from the corresponding author on reasonable request.
