# Supplementary material for: Cannabinoid receptor 1 expression is higher in muscle of old vs. young males, and increases upon resistance exercise in older adults
Source: Sci Rep. 2021 Sep 15;11:18349. doi: 10.1038/s41598-021-97859-3 (PMC8443742; doi:10.1038/s41598-021-97859-3)
Supplement: Supplementary file 1 — Supplementary Figures. [file 41598_2021_97859_MOESM1_ESM.docx]

Cannabinoid receptor 1 expression is higher in muscle of old vs. young males, and increases upon resistance exercise in older adults

Sebastiaan Dalle, PhD & Katrien Koppo*, PhD

Exercise Physiology Research Group, Dept. of Movement Sciences, KU Leuven, Tervuursevest 101, 3001 Leuven, Belgium

ORCID IDs: SD: 0000-0001-5734-3993; KK: 0000-0002-6022-1097

*corresponding author: katrien.koppo@kuleuven.be; +32 16 37 26 80


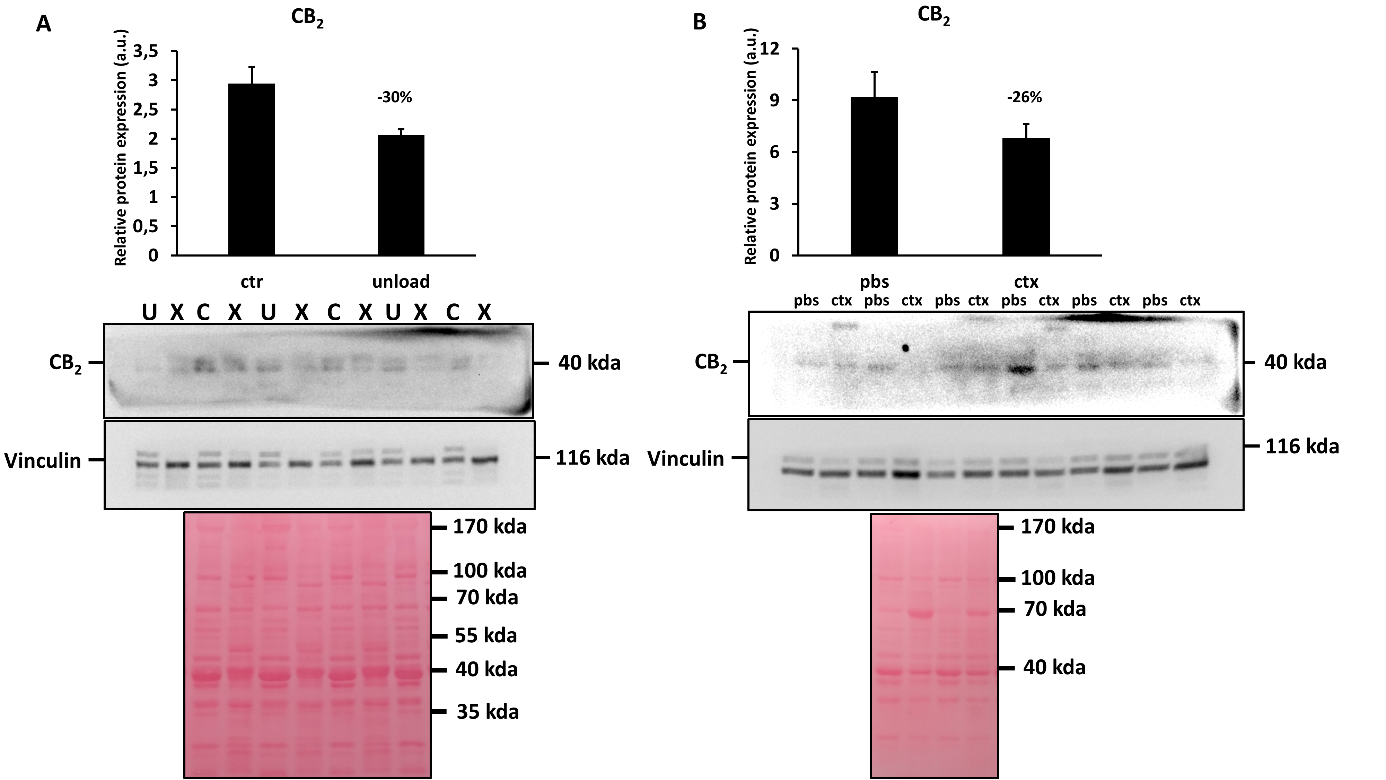


Supplementary figure 1: Cannabinoid receptor 2 (CB_2_) expression is decreased in the *m. Gastrocnemius* of mice after 10 days of muscle unloading (U) through hind limb suspension compared to free-living controls (Ctr/C) (A) and in the *m. Tibialis Anterior* after cardiotoxin-injected muscle injury (ctx) compared to PBS-injected controls (pbs). X lanes in Suppl. Fig. 1A refer to lanes in which CBR2 expression in heart muscle tissue is detected, irrelevant to the context of the present study.


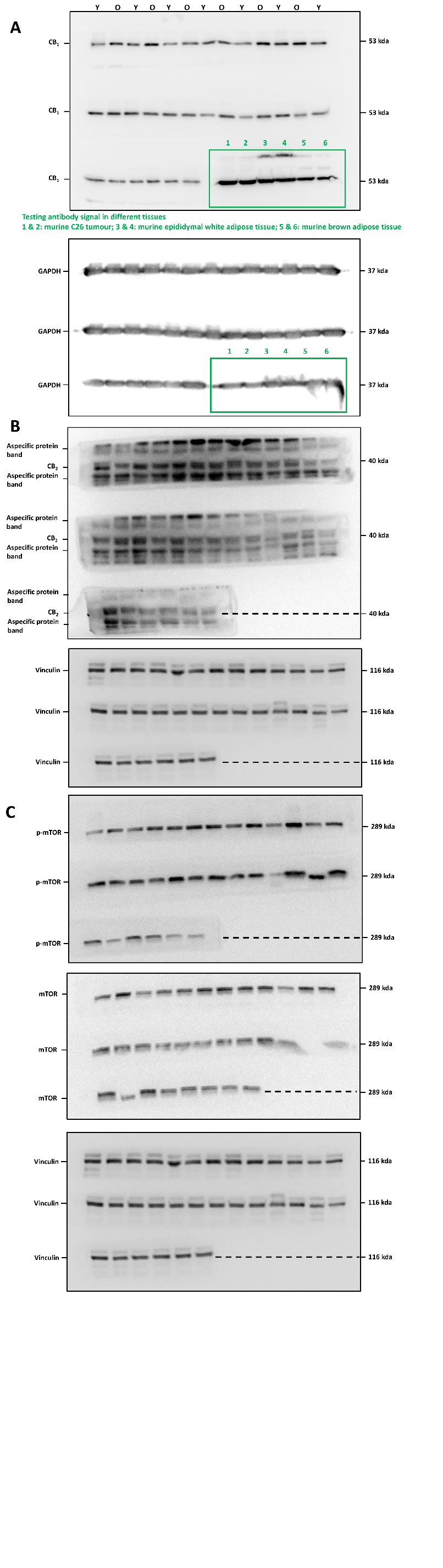

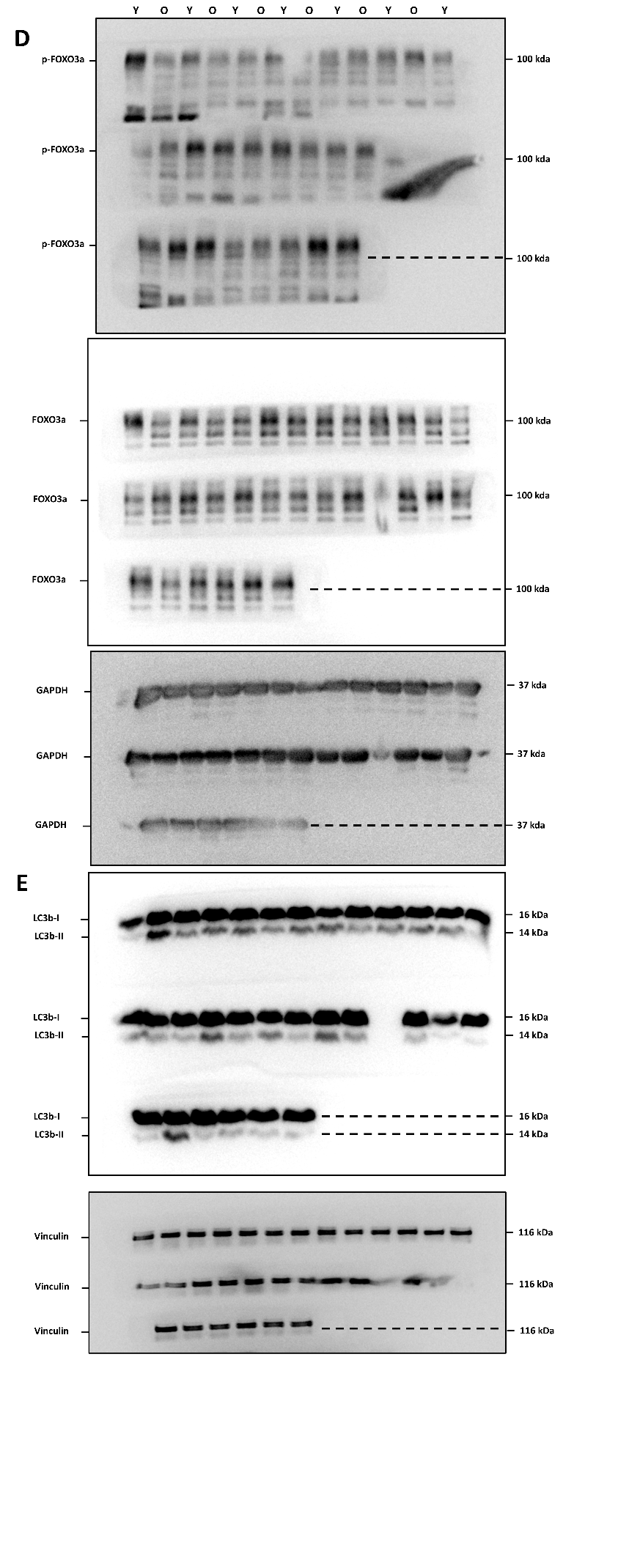

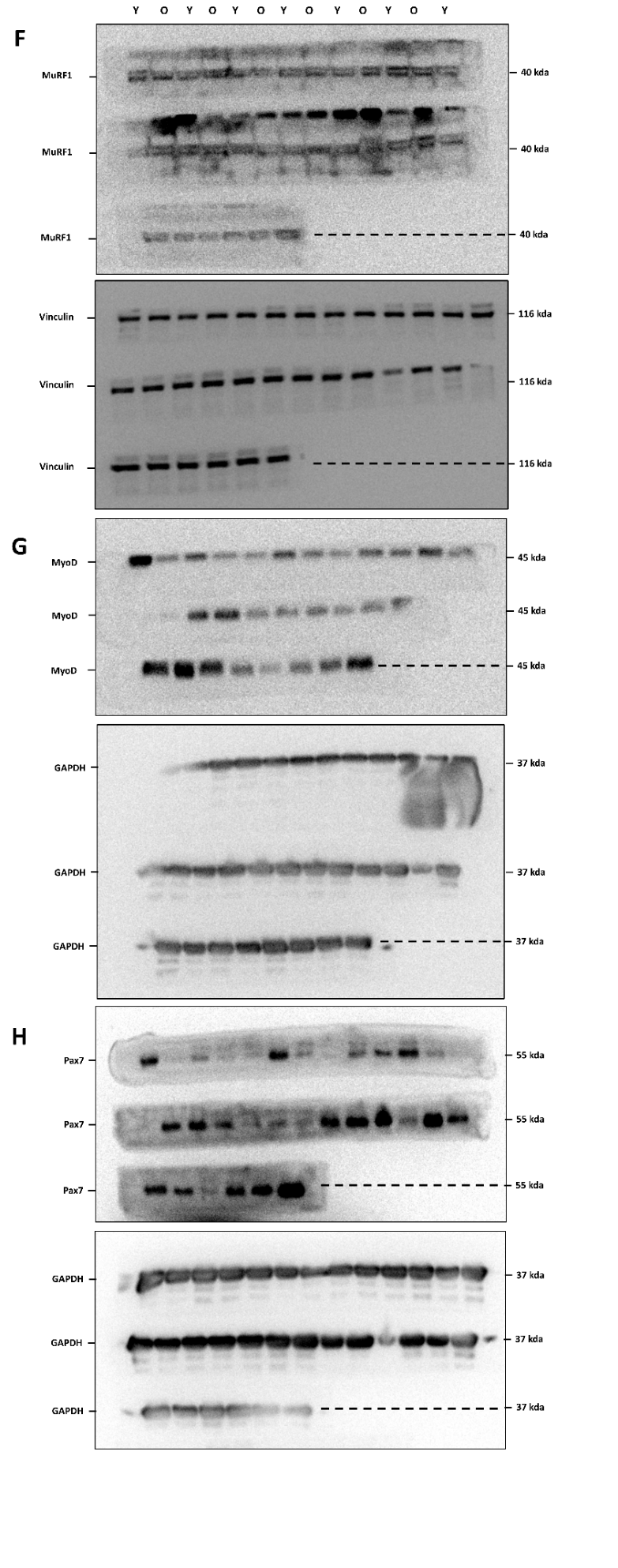


Supplementary figure 2: The full-length western blot membrane output of the blots displayed in Figure 1. Differences in the protein expression of cannabinoid receptor 1 (CB_1_, panel A), CB_2_ (panel B) and phospho and total mammalian target of rapamycin (mTOR) (at Ser2448) (panel C), phospo and total forkhead box O (FOXO3a) (at Thr32) (panel D), microtubule-associated protein 1A/1B-light chain 3 (LC3b) (panel E), muscle RING finger-1 (MuRF1) (panel F), MyoD (panel G) and Pax7 (panel H) between skeletal muscle of young (Y) and old (O) adults. Protein targets were normalized to glyceraldehyde 3-phosphate dehydrogenase (GAPDH), vinculin or the total form. Red boxes denote to the regions of the blots that are used in the main figures.


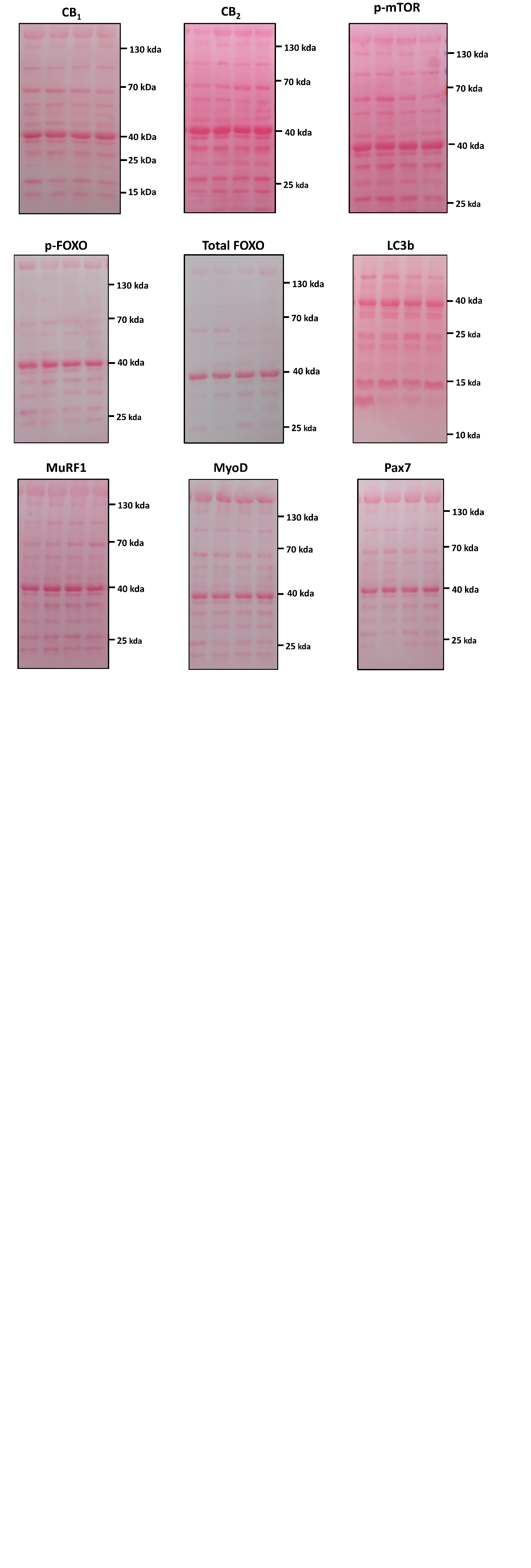


Supplementary figure 3: Ponceau red staining of the representative lanes of the western blot analyses presented in Figure 1. The first and third lane refer to the total protein content in the muscle of old adults and the second and fourth lane refer to the total protein content in the muscle of young adults. mTOR: mammalian target of rapamycin; FOXO: forkhead box O; LC3b: microtubule-associated protein 1A/1B-light chain 3; MuRF1: muscle RING finger-1 (MuRF1).


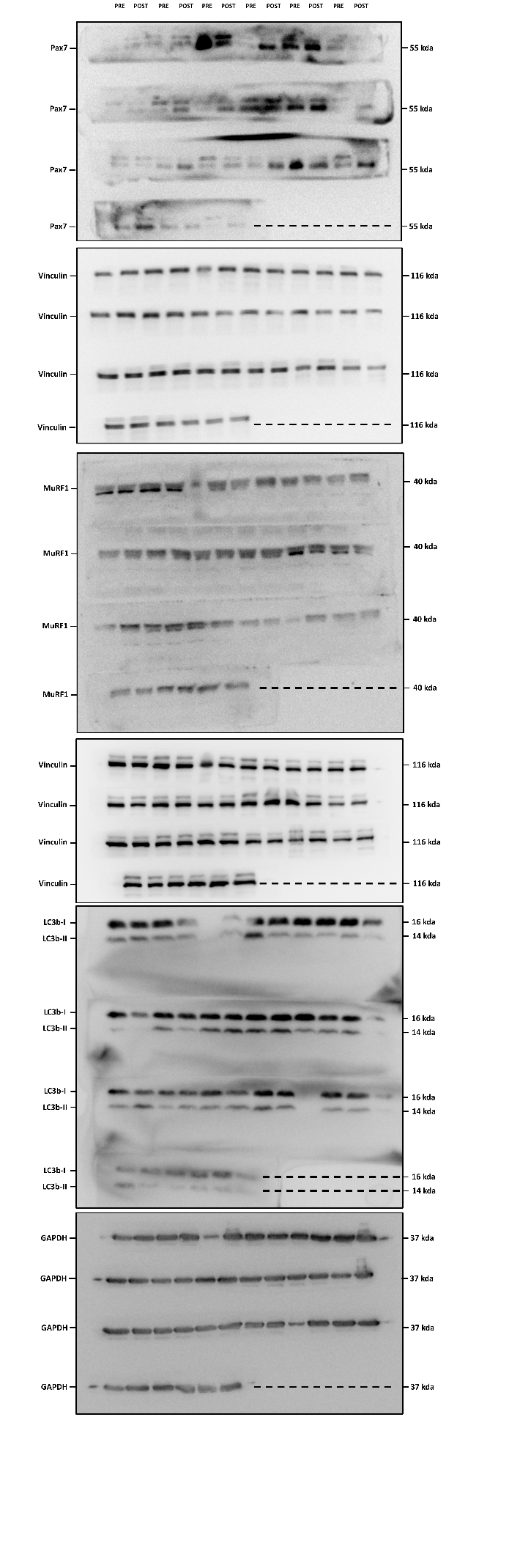

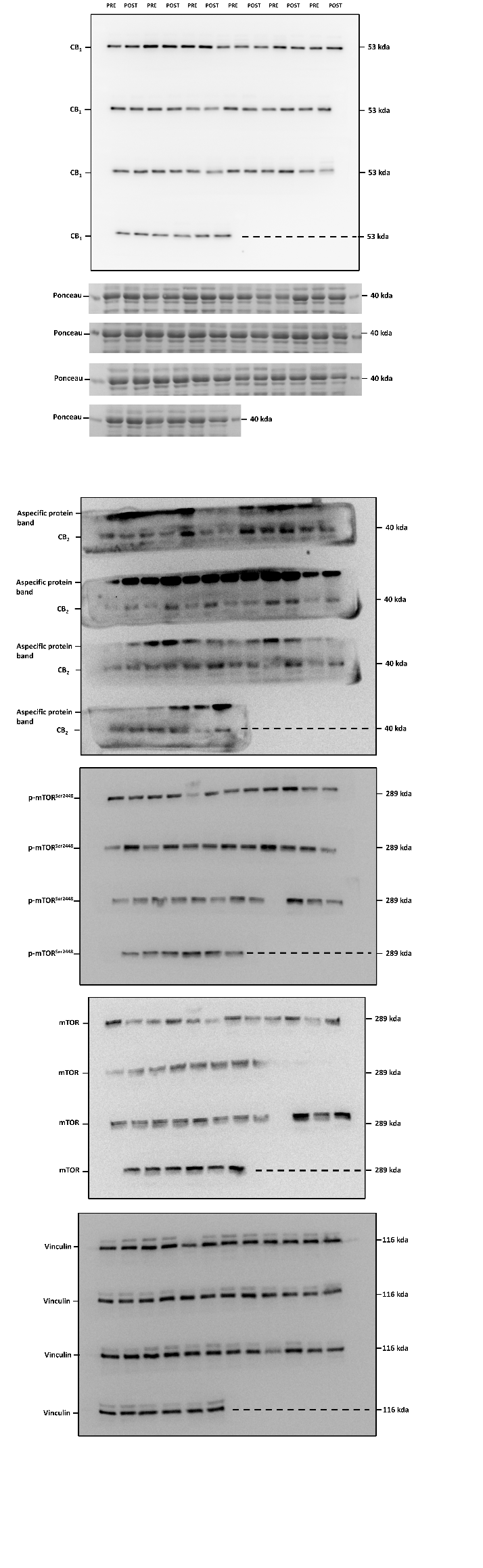

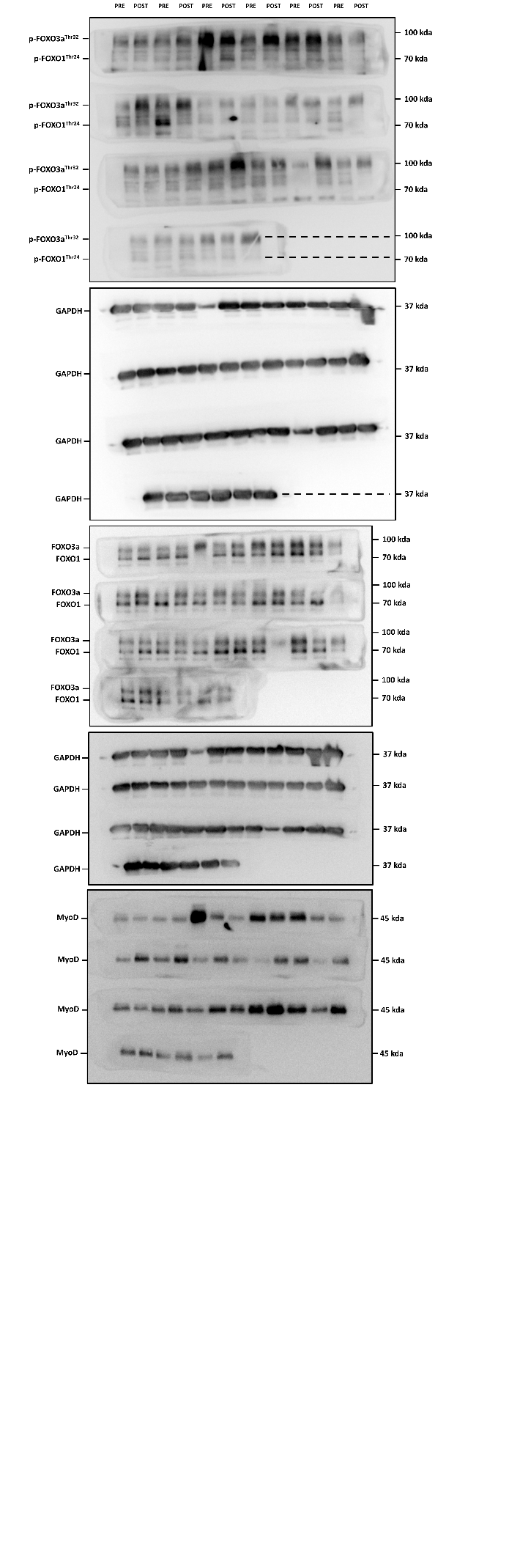


Supplementary figure 4: The full-length western blot membrane output of the blots displayed in Figure 2. Differences in the protein expression of cannabinoid receptor 1 (CB_1_), CB_2_, phospho-mammalian target of rapamycin (p-mTORSer2448), p-forkhead box O (FOXO)1Thr24/3aThr32, total FOXO1/3a, microtubule-associated protein 1A/1B-light chain 3 (LC3b), muscle RING finger-1 (MuRF1), MyoD and Pax7 before (PRE) and after (POST) resistance exercise in old adults. Protein targets were normalized to glyceraldehyde 3-phosphate dehydrogenase (GAPDH), vinculin or the total form. The fifth and the sixth lane of the first membrane (Pre and Post sample of the same participant), and the ninth and the tenth lane of the third membrane (Pre and Post sample of the same participant) are not included in the analyses, as lane 5 of membrane 1 and lane 9 of membrane 3 mainly contains intermuscular fat tissue and not muscle tissue. Red boxes denote to the regions of the blots that are used in the main figures.

Supplementary figure 5: Ponceau red staining of the representative lanes of the western blot analyses presented in Figure 2. The first and third lane refer to the total protein content prior to the 12-wk resistance exercise protocol in 2 old adults and the second and fourth lane refer to the total protein content after the exercise protocol in the respective old adults. FOXO: forkhead box O; LC3b: microtubule-associated protein 1A/1B-light chain 3; MuRF1: muscle RING finger-1 (MuRF1).
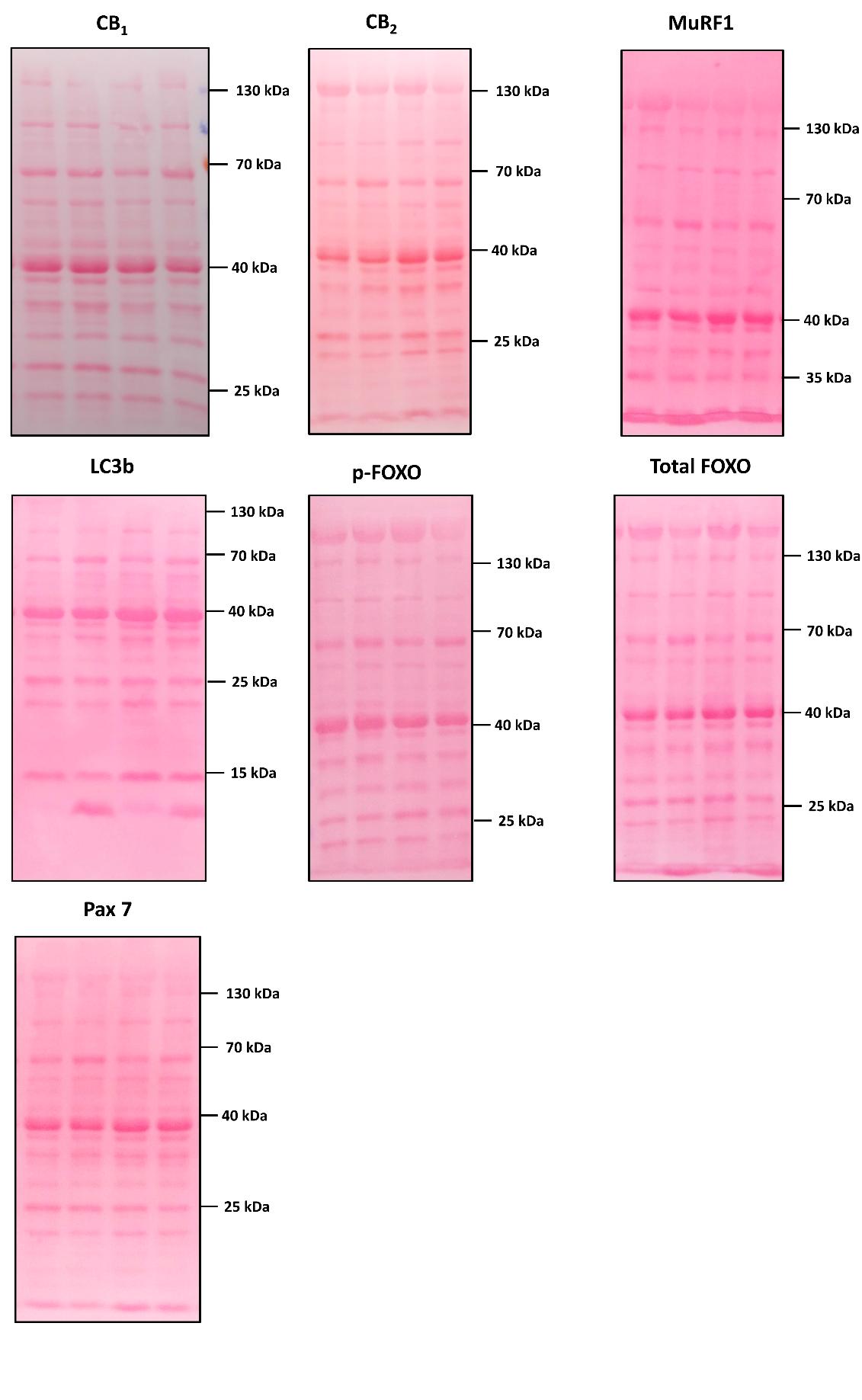


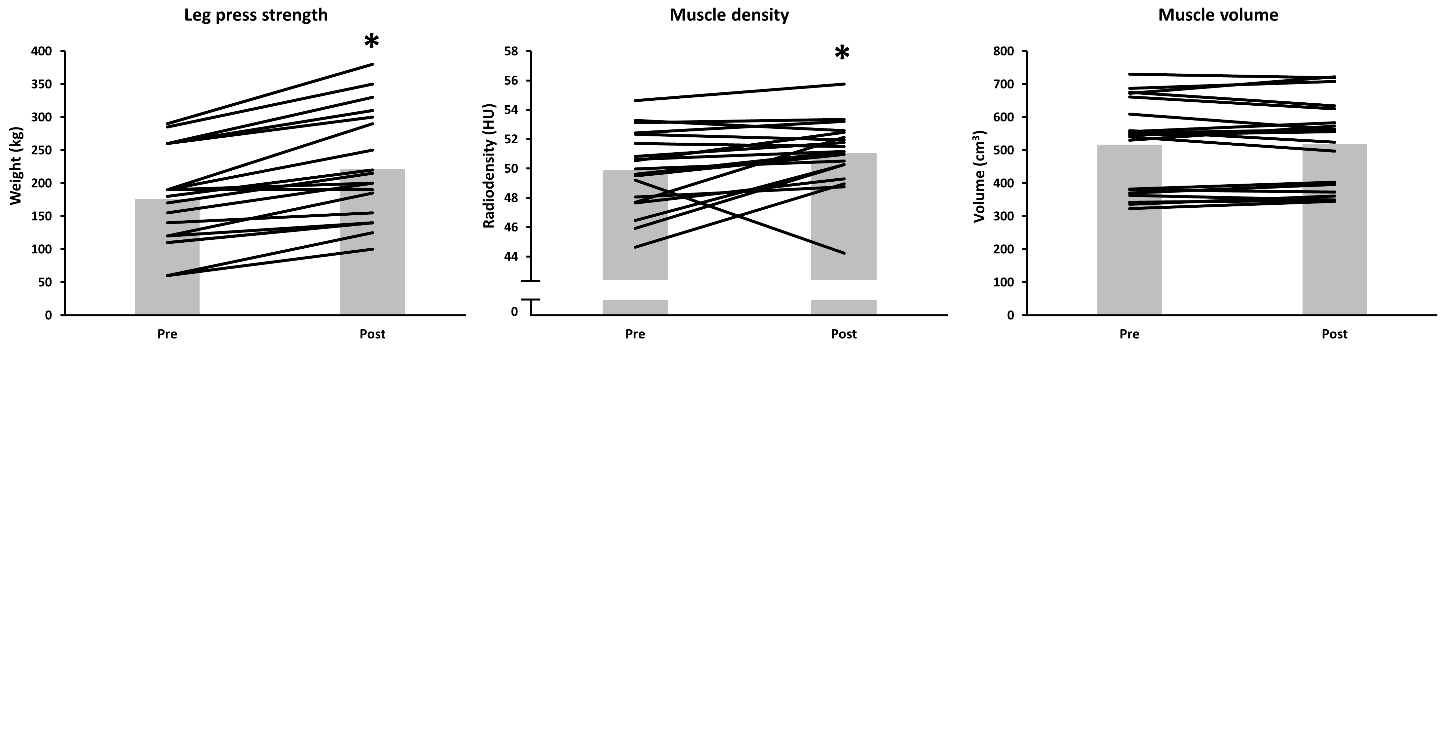

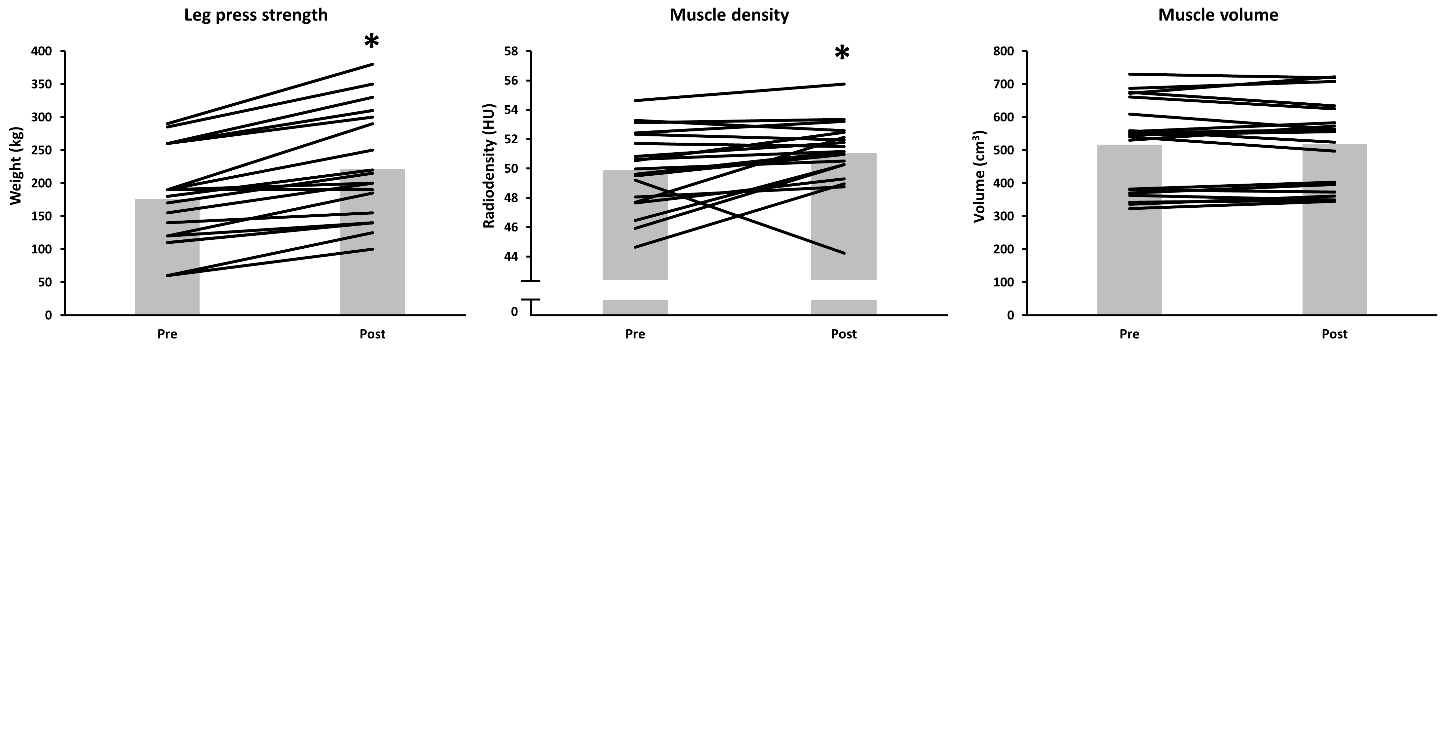

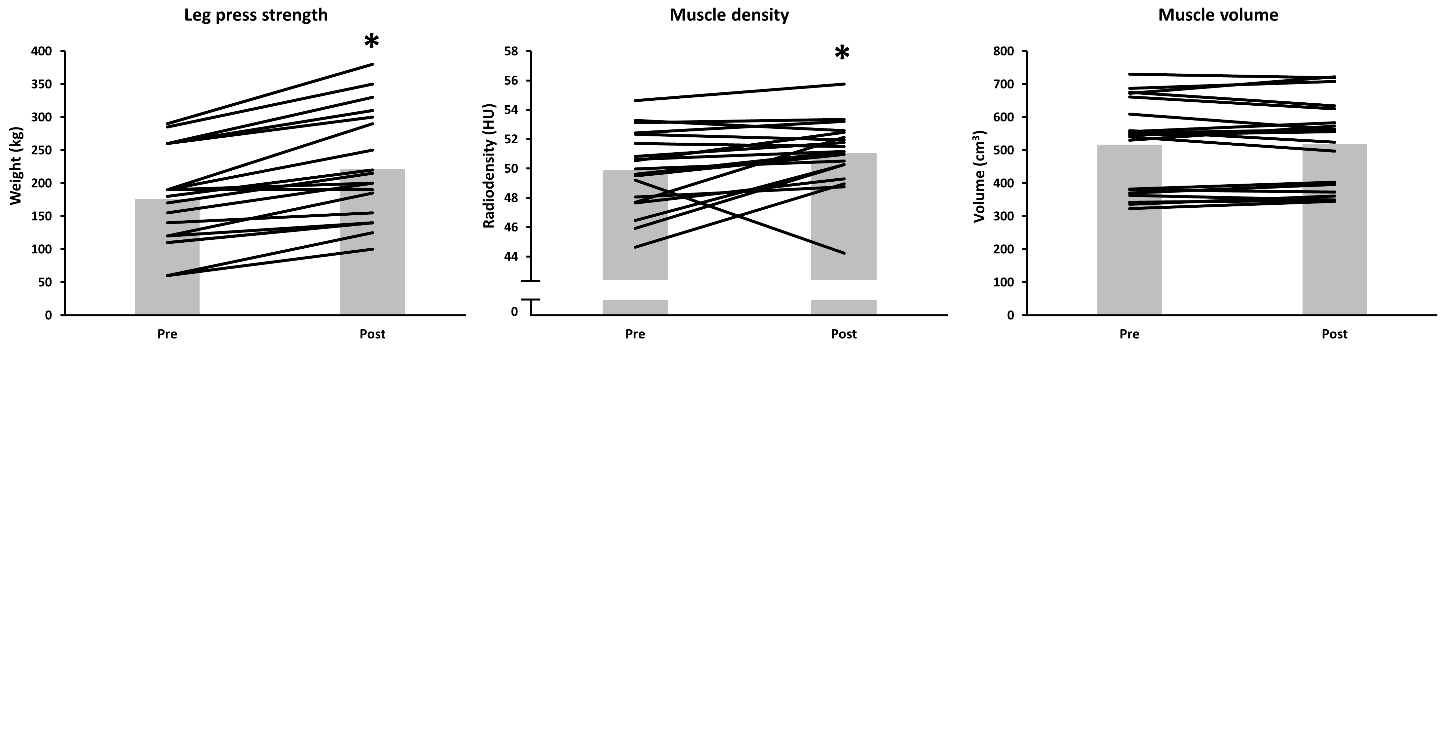

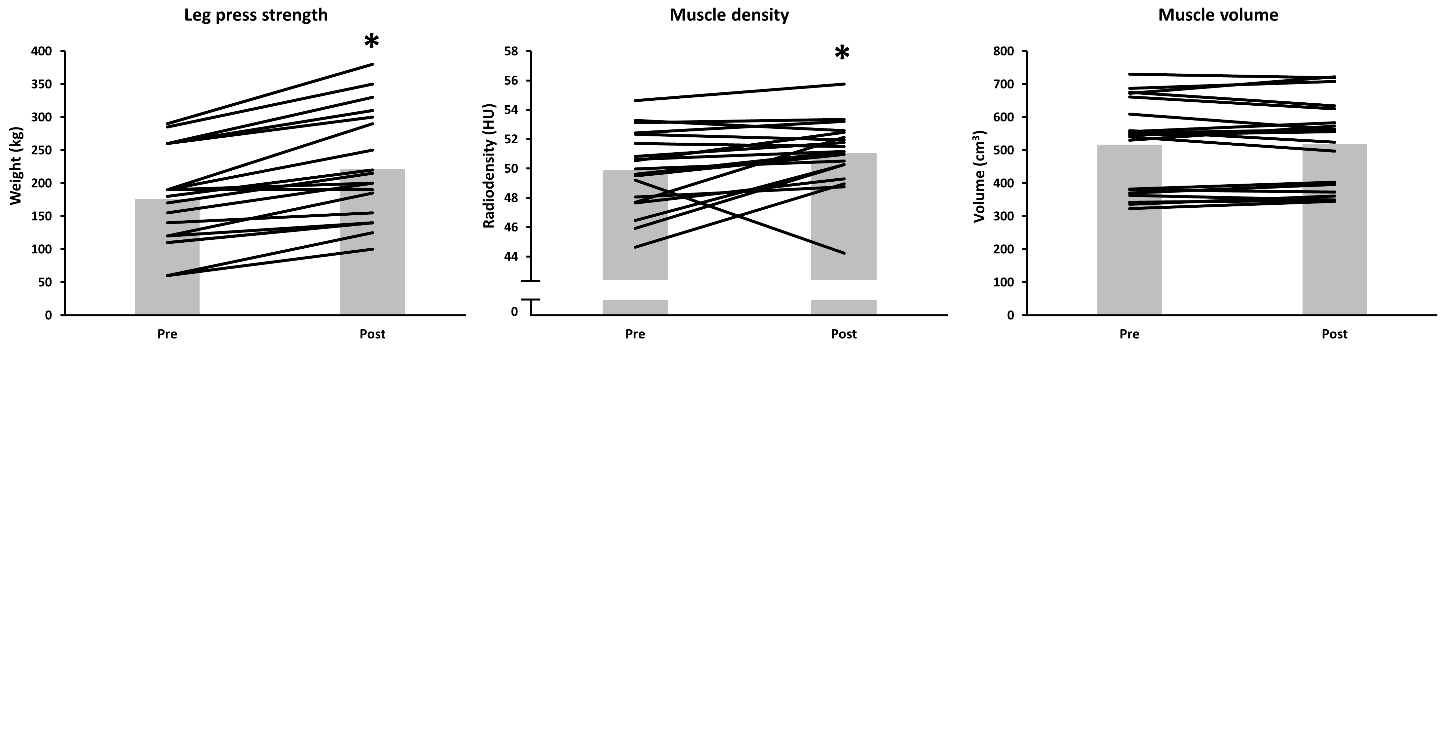

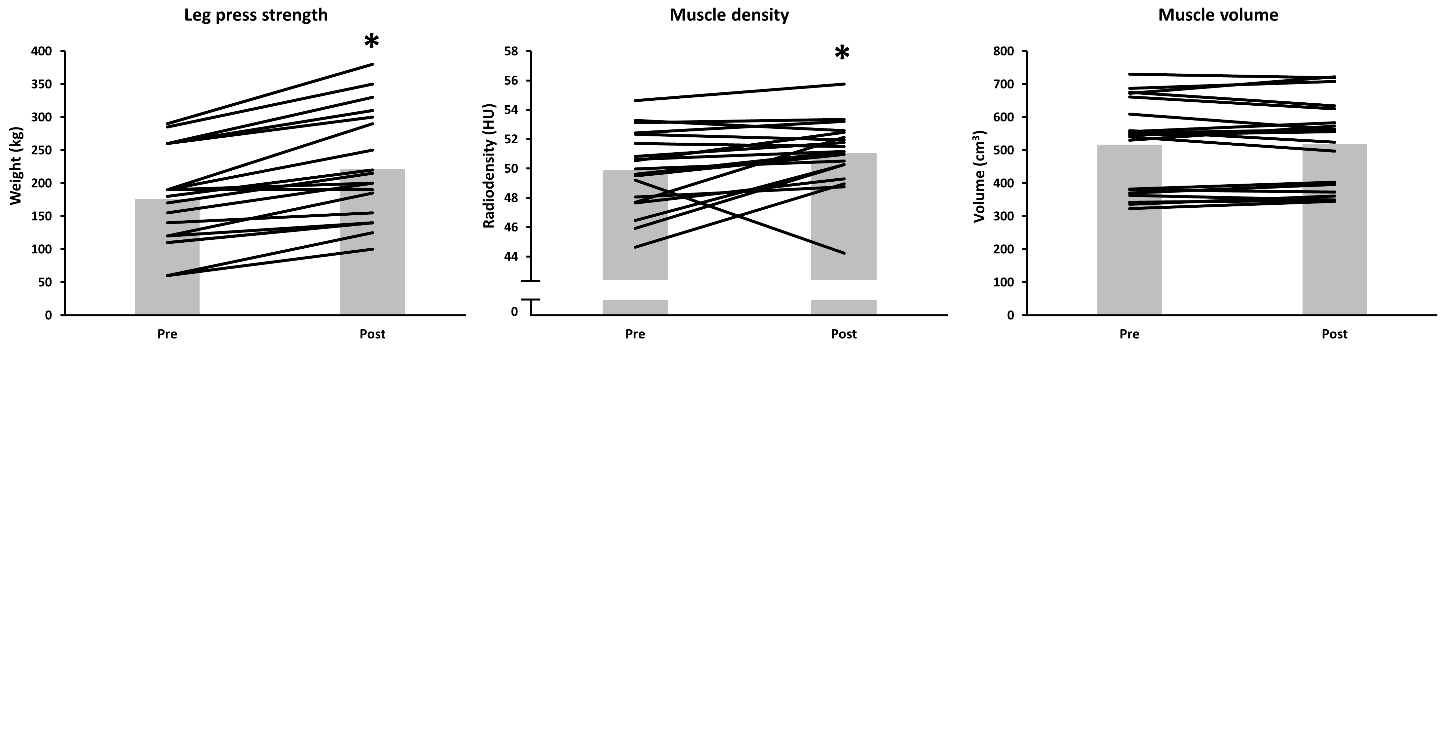

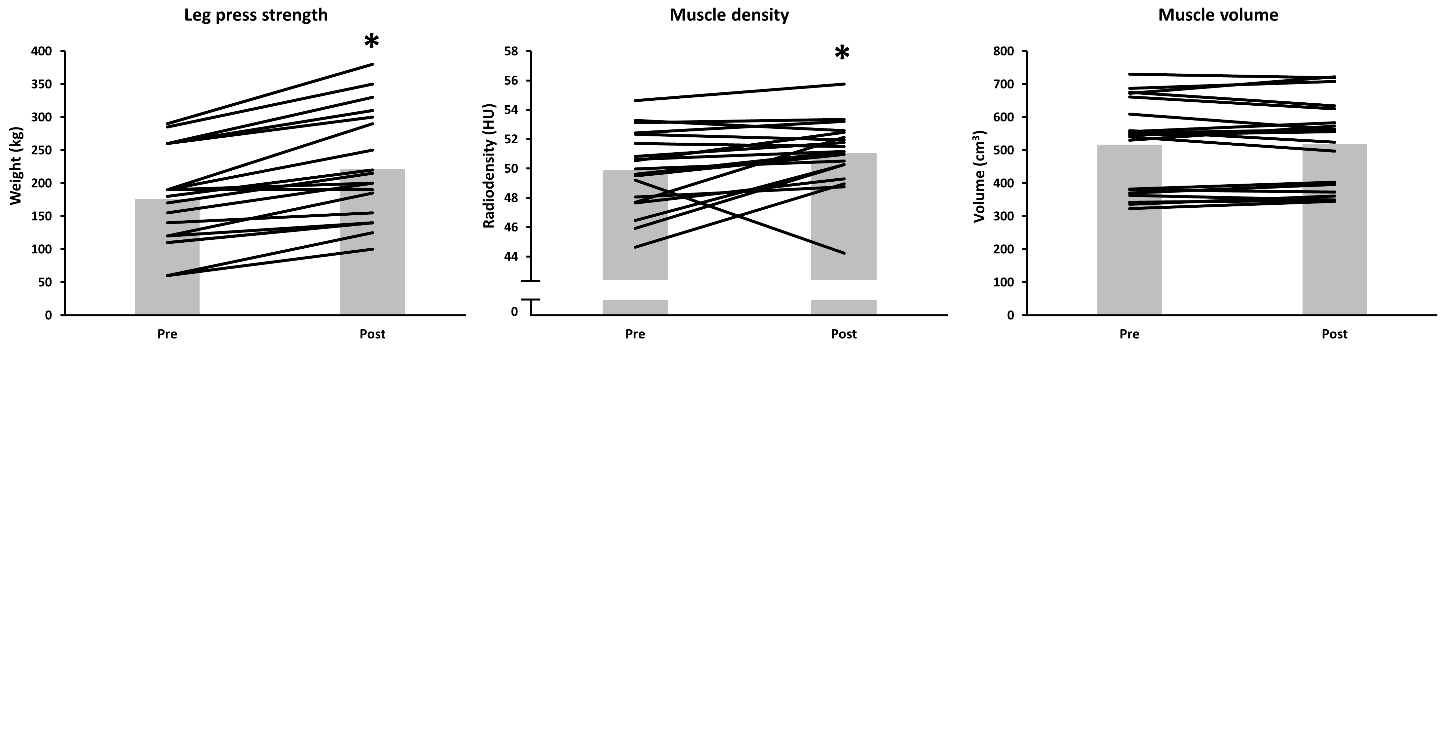

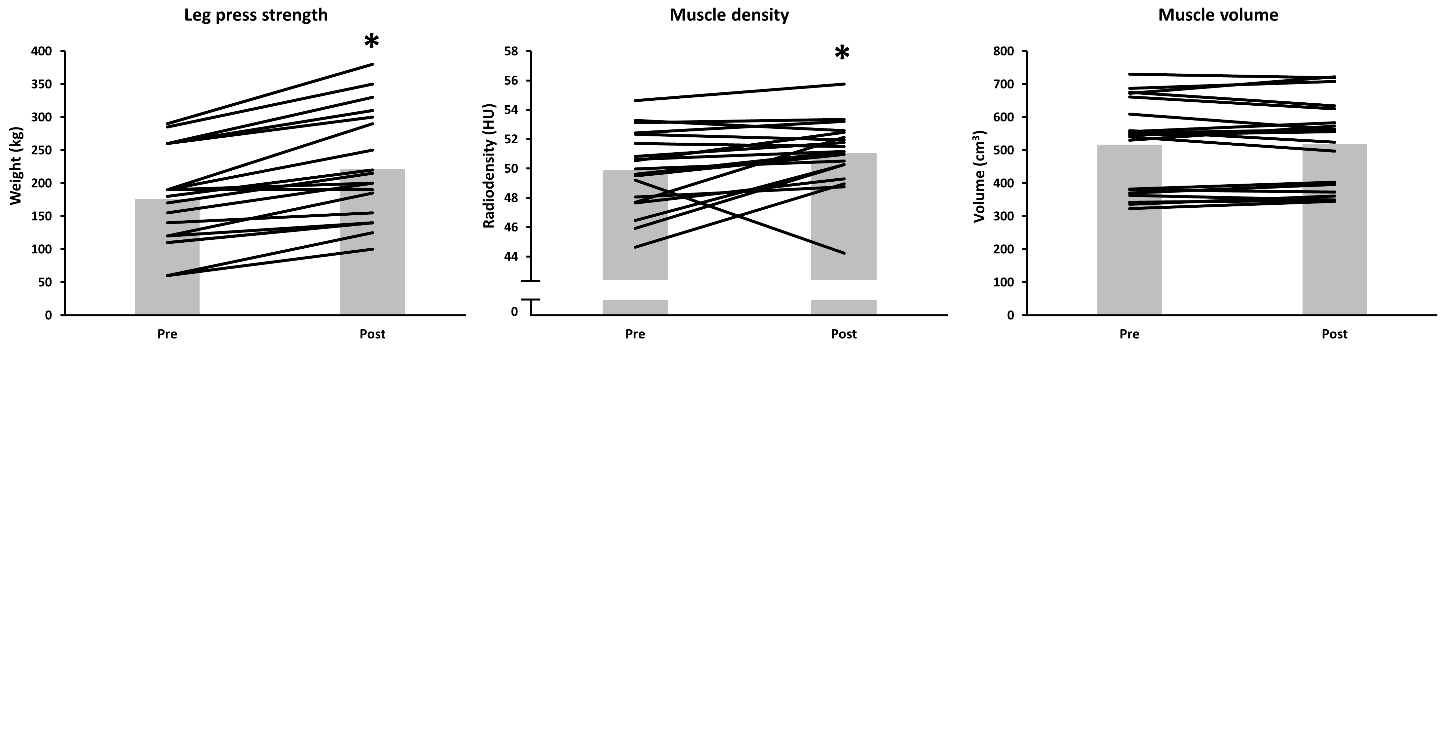

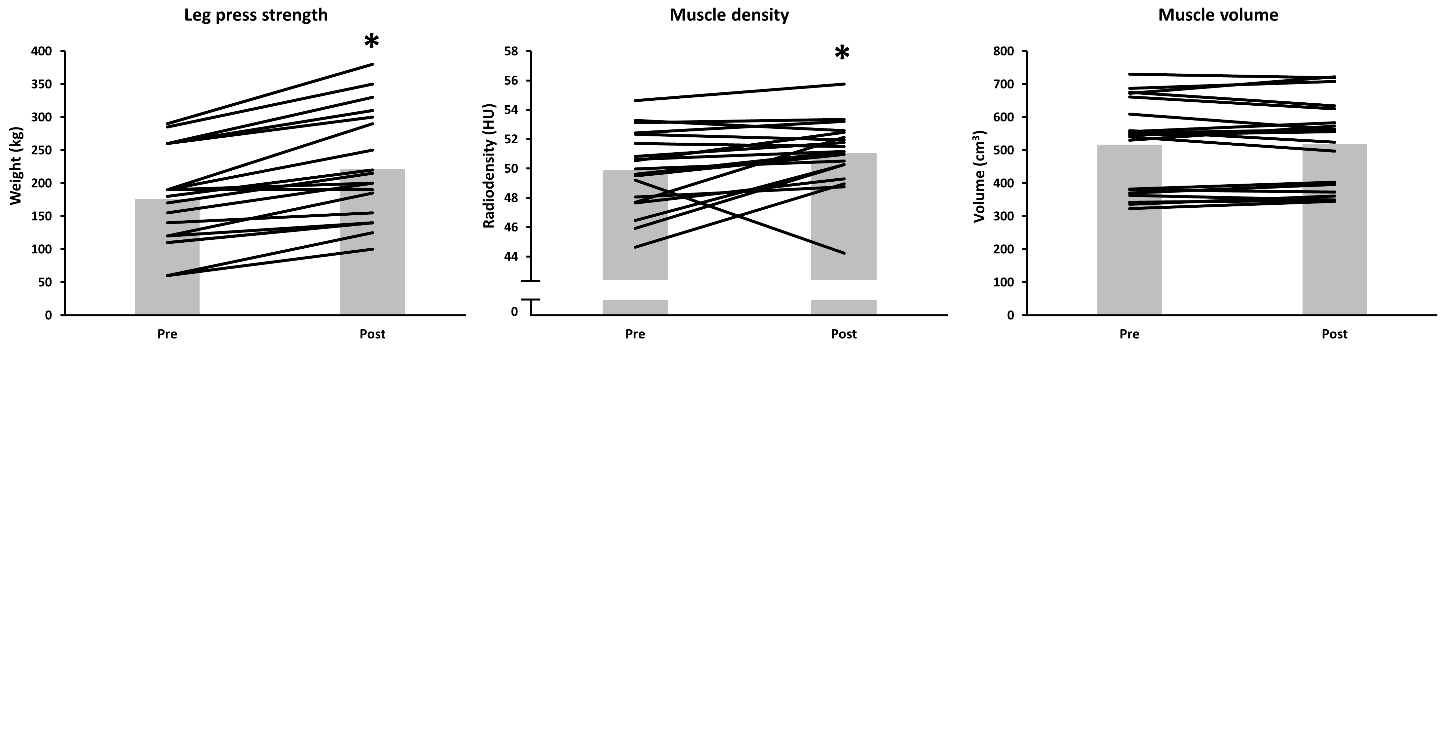


Supplementary figure 6: Maximal (1-repetition maximum) muscle strength, upper leg muscle density (Hounsfield Unit, HU) and upper leg muscle volume prior to (Pre) and following (Post) the 12-week training intervention in 19 older adults (65-78yr). *p<0.05 for Paired-Samples T-Test.
